# Formulation of Ethyl Cellulose Microparticles Incorporated Pheophytin A Isolated from *Suaeda vermiculata* for Antioxidant and Cytotoxic Activities

**DOI:** 10.3390/molecules24081501

**Published:** 2019-04-17

**Authors:** Hamdoon A. Mohammed, Mohsen S. Al-Omar, Mahmoud Zaki El-Readi, Ahmad H. Alhowail, Maha A. Aldubayan, Ahmed A. H. Abdellatif

**Affiliations:** 1 Department of Pharmacognosy, Faculty of Pharmacy, Al-Azhar University, Cairo 11371, Egypt; 2 Department of Medicinal Chemistry and Pharmacognosy, College of Pharmacy, Qassim University, 51452 Al Qassim, Kingdom of Saudi Arabia; m.omar@qu.edu.sa; 3 Department of Biochemistry, Faculty of Pharmacy, Al-Azhar University, Assiut 71524, Egypt; mzreadi@uqu.edu.sa; 4 Department of Clinical Biochemistry, Faculty of Medicine, Umm Al-Qura University, Abdia, Makkah 21955, Saudi Arabia; 5 Department of Pharmacology and Toxicology, College of Pharmacy, Qassim University, 51452 Al Qassim, Kingdom of Saudi Arabia; aalhowail@qu.edu.sa (A.H.A.); m.aldubayan@qu.edu.sa (M.A.A.); 6 Department of Pharmaceutics, College of Pharmacy, Qassim University, 51452 Al Qassim, Kingdom of Saudi Arabia; a.abdellatif@qu.edu.sa; 7 Department of Pharmaceutics and Industrial Pharmacy, Faculty of Pharmacy, Al-Azhar University, Assiut 71524, Egypt

**Keywords:** *Suaeda vermiculata*, pheophytin a, polymeric microparticles, ethyl cellulose, antioxidant activity

## Abstract

Background: This study is designed to discover a method for delivering an efficient potent pheophytin a (pheo-a) into more absorbed and small polymeric ethyl cellulose (EC) microparticles. Methods: Silica gel and Sephadex LH-20 columns were used to isolate pheo-a from the chloroform extract of the edible plant, *Suaeda vermiculata*. Pheo-a was incorporated into EC microparticles using emulsion-solvent techniques. The antioxidant activity of pheo-a microparticles was confirmed by the level of superoxide radical (SOD), nitric oxide (NO), and reducing power (RP) methods. Meanwhile, the cytotoxic effect of the product was investigated on MCF-7 cells using MTT assay. Results: Pheo-a was isolated from *S. vermiculata* in a 12% concentration of the total chloroform extract. The structures were confirmed by NMR and IR spectroscopic analysis. The formulated microparticles were uniform, completely dispersed in the aqueous media, compatible as ingredients, and had a mean diameter of 139 ± 1.56 µm as measured by a particle size analyzer. Pheo-a demonstrated a valuable antioxidant activity when compared with ascorbic acid. The IC_50_ values of pheo-a microparticles were 200.5 and 137.7 µg/mL for SOD, and NO respectively. The reducing power of pheo-a microparticles was more potent than ascorbic acid and had a 4.2 µg/mL for IC_50_ value. Pheo-a microparticles did not show notable cytotoxicity on the MCF-7 cell line (IC_50_ = 35.9 µg/mL) compared with doxorubicin (IC_50_ = 3.2 µg/mL). Conclusions: the results showed that water-soluble pheo-a microparticles were prepared with a valuable antioxidant activity in a wide range of concentrations with a noteworthy cytotoxic effect.

## 1. Introduction

*Suaeda vermiculata* is a halophytic plant that belongs to family *Chenopodiaceae*. The plant is widely distributed in the arid areas of the desert where water is rare and high salinity is the predominant environment. Therefore, *S. vermiculata* is widely spread in Arabian Gulf countries and is considered to be one of the most widespread plants in central Saudi Arabia, especially the Qassim region [1]. *S. vermiculata* is an edible plant and Bedouins use it as an appetizer for their domestic animals, such as camels and sheep [2]. Like other halophytes, *S. vermiculata* contains enzymatic and/or non-enzymatic antioxidant mechanisms that enable such plants to survive in a high saline condition. Strong scavenging activity against the 2,2-diphenyl-1-picrylhydrazyl (DPPH) free radical has been reported for *S. vermiculata* extracts and essential oils [3,4]. *S. vermiculata* extracts also have the ability to chelate metals, e.g., Iron. [4,5].

**Figure 1:**
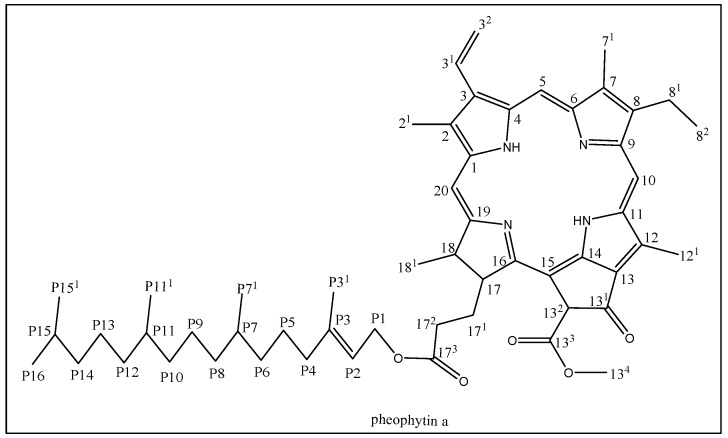
Chemical structure of pheo-a.

Chlorophylls which constitute the most abundant pigments in plants, play a major role in the plant and algae photosynthesis processes. Chlorophylls a and b, and their de-magnesium analogues, pheophytin a (pheo-a) and pheophytin b, respectively, are common chlorophylls in plant [6]. Pheo-a is a porphyrin macro-cyclic derived molecule consisting of four pyrrole rings connected together through methylene bridge with a phytyl side change linked to the macocyclic structure at carbon 17^3^ through ester linkage [7] (Figure 1). Pheo-a is a dietary chlorophyll present in the edible green plants and algae and constitute the main pigment present in known healthy, edible plants, e.g., the olive oil color is mainly due to pheo-a [8]. In addition, virgin olive oil withstands oxidation due to the presence of pheo-a [9]. Biological activities, such as anti-inflammatory [10], antimicrobial [11], antigenotoxic [12], and anti-hepatitis C virus [13], have been reported for the pheo-a compound.

Furthermore, the method of encapsulation of pheo-a has several advantages such as increasing the permanence and stability of pheo-a via their shielding against moisture and atmospheric oxidation and masking its disagreeable taste [14]. Additionally, it can enhance the surface area of chemically incompatible substances, and thus reduce the gastrointestinal adverse effects of pheo-a [15]. It was reported that the ferrous sulphate and potassium chloride solutions have been chemically solubilized through the modification of dissolution rates and the duration of action via encapsulation processes [16,17,18]. Ethyl cellulose (EC) is a type of cellulosic polymer that was used in this study to formulate pheo-a microparticles with high water solubility and stability in pH 7.1 for further usage as a formulated dosage form. EC is an inert, hydrophobic polymer and has been extensively used as a release rate controlling material. EC is soluble in organic solvents, such as acetone, methanol, ethanol, and toluene; and insoluble in water, glycerin, and propylene glycol. EC is the most stable form of cellulose derivatives and is used in many pharmaceutical preparations [19]. The pheo-a was formulated as microparticles, using a solvent evaporation method for water dispensable microparticles. This process is considered to be a method for the core material and polymer to be dissolved or dispersed in a water-immiscible volatile organic solvent (casting solvent). Furthermore, the resulting solution or dispersion was emulsified in an aqueous medium to form the micro-droplets from pheo-a. These microparticles tend to be a solidified filtrated form as a product of free flowing microparticles [20].

Practically speaking, pheo-a is a water insoluble material that can’t be used as a pure compound for a patient’s treatment. The encapsulation of pheo-a with EC was used to enhance the solubility and spreadability of the final product, and hence to decrease its side effects on the stomach due to ethyl cellulose requires a pH above 7 for release. Furthermore, this ethyl cellulose polymer is cheap, safe, and available [21,22].

The present study is related to the isolation of the pheo-a compound from an edible halophytic plant, *S. vermiculata*. Moreover, the pheo-a was formulated in the form of EC micro particles to enhance the poor solubility of the parent drug and also to improve the antioxidant and cytotoxic activities of pheo-a against known antioxidant agents, ascorbic acid, and the cytotoxic agent, doxorubicin.

## 2. Results and Discussion

### 2.1. Isolation and Structure Elucidation of Pheo-a

The chromatographic and spectroscopic techniques were used to isolate and identify pheo-a in a pure form. The major pigments containing chloroformic sub-fractions have been determined using thin layer chromatographicy (TLC). TLC indicated, moreover, that sub-fractions were abundant with one spot pigment in addition to ultraviolet (UV) inactive steroidal components. Further, sephadex LH-20 (gel chromatographic column) has been used to isolate the pigment as a major compound (pheo-a), which was first eluted by methanol. The pheo-a was further purified by a silica gel column using *n* hexan: an ethyl acetate as an eluent. The sephadex column also isolated pure steroidal compounds (β-sitosterol) in addition to a mixture of steroids that were eluted all together. The IR spectrum of pheo-a exhibited absorptions at 2922 (C-sp3), 3435 (NH), 1694 (CO) and 1377 cm^−1^ (CN); these results are consistent with the presence of a porphyrin nucleus in this compound [23]. The proton nuclear magnetic resonance (^1^H-NMR) spectrum of pheo-a showed characteristic peaks for signals of the porphyrin skeleton and pheophytin a, as the spectrum appears at δ_H_ 3.17, 3.40, and 3.67 ppm for three singlet methyl at position number 7, 2, and 12, respectively. Sharp singlets at δ_H_ 9.50, 9.15, and 8.90 ppm were assigned for the highly downfield protons at the 5, 10, and 12 positions, respectively (Figure 1). The quartet signal at δ_H_ 3.68 ppm and triplet signal at δ_H_ 1.70 ppm was assigned to the 2H-81 and 3H-82, respectively. The ^13^C-NMR spectrum has been used to confirm the pheophytin a structure. Three carboxy groups were resonating at δ_C_ 189.8, 173.1, 172.4 ppm for C-13^1^, C-13^3^, and C-17^3^, respectively. The methyl ester peak appeared as a quartet carbon at δ_C_ 53 ppm with three protons resonating at δ_H_ 3.89 ppm; this peak was similar to that for the pheophytin-a methyl ester reported in the literature [24]. Phytyl ester moiety has been identified and compared to the data published for pheophytin a [24,25]. According to spectral data, in addition to literature, pheo-a has been isolated for the first time from *S. vermiculata* (Figure 1). The NMR and FTIR spectra for pheo-a and compound 2 are shown in the Supplementary Materials.

### 2.2. Size and Weight of Ethyl Cellulose Microparticles

**Figure 2:**
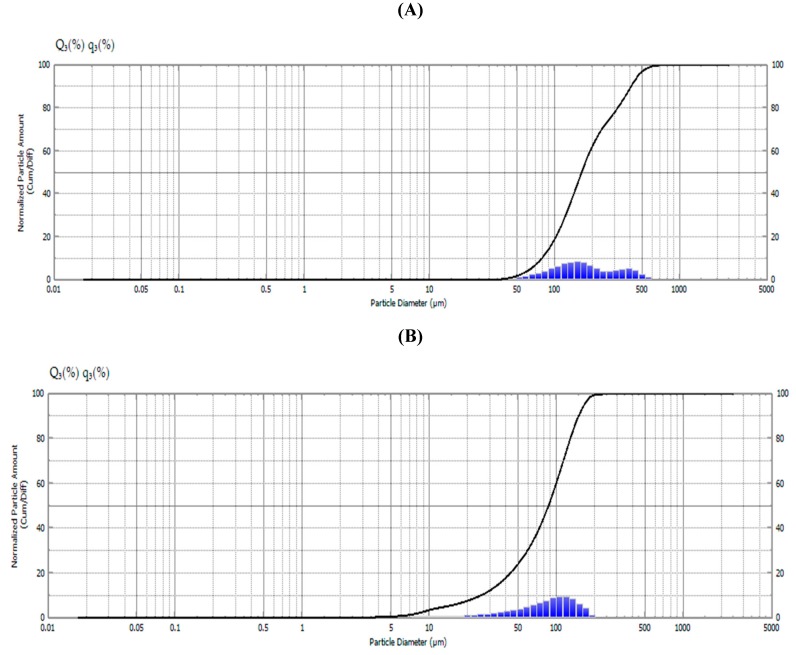
The decrease of the particle size from 163 µm for plain ethyl cellulose (EC) microparticles (**A**) to 139.23 µm after the formation of EC-Pheo-a (**B**).

Pheo-a EC microparticles were formulated to give a stable suspended solution. The system showed no visible coalescence. Collectively, these results indicated a significant difference from phoe-a itself. Pheo-a has no solubility and is completely immiscible in water. However, the formulated microparticles are completely dispersed in water. The entrapment of pheo-a in the EC particles was confirmed by changing the EC particles to a green color, the color of pheo-a. The particles size was decreased from 163 ± 1.5 µm for plain EC (Figure 2A) to microparticles 139.23 ± 0.8 µm after the formation of EC- pheo-a (Figure 2B); this result is due to the uniformity of the formulated particles, which showed only one peak. The plain microparticles were heterogenous and showed two different peaks, while the microparticles that loaded pheo-a showed homogeneity in the formulation in the form of only one peak and without any aggregation. The obtained result is considered an intensity-weighed value and a sensitive method for the formulated larger size types. This result may be mainly important for aggregated or adulterated models but is also of concern for broadly non-homogeneous examples. For evaluation, the plain EC-microparticles and the pheo-a microparticles were used as size standards with a typical size distribution, which indicates a homogeneous sample [26,27,28]. After purification, the particles were found to be stable without aggregation, and the formulated particles were found also to be reasonably stable in size and didn´t form aggregates. The microparticles were spherical in shape and had a particle size of ≈163–149 µm.

### 2.3. Infrared Fourier Transform Spectroscopy Analysis (FTIR)

**Figure 3:**
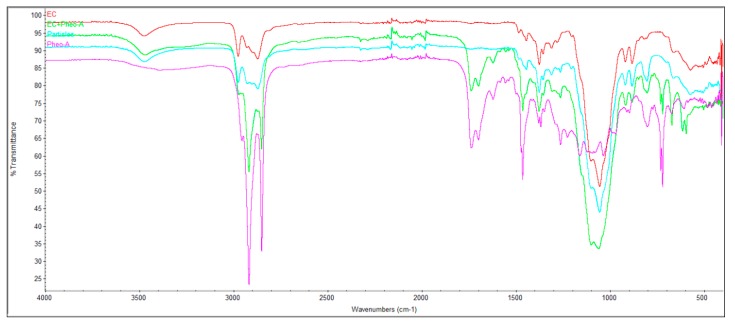
Infrared fourier transform spectroscopy analysis (FTIR) spectra using attenuated total reflectance method at room temperature. From top to bottom: EC, EC+Pheo-a, the formulated EC-pheo-a, and physical mixture of EC & pheo-a.

The main bands of pheo-a, EC, and the physical mixture of EC & pheo-a spectra of the dried samples are shown in Figure 3. The corresponding spectra were smoothened and normalized in order to define the most important bands. The FTIR spectrum of pheo-a showed absorption at 2923 (C-H of phytol) and 2850 cm^−1^ for the anti-symmetric and symmetric C-H stretching of the methylene groups, respectively [29]. The FTIR peaks of 3390 (N-H), 1736 (esters, COOR), 1705 (ketone, C=O), 1617 (C=C), 1458 (C-H scissoring), 1250 (C-N), 1150 (bridges of C-O-C) can be anticipated for the pheo-a structure [29,30]. FTIR analysis was performed in order to detect the unwanted physical interaction of the pheo-a and EC. If FTIR showed a broad peak in the unexpected area, which means that EC and pheo-a interacted together to form a physical complex which indeed prevented the release of pheo-a from the formulated EC-microparticles. FTIR showed no differences in the shape and position of peaks between pheo-a, EC and the physical mixture of both, which means that there is no interaction between pheo-a and EC; thus, pheo-a is still free and can be released easily from EC microparticles. FTIR showed the characteristic fingerprint of pheo-a and EC, which proved that two covalent bonds appeared in physical mixture of both [15,21].

### 2.4. Superoxide Radical Scavenging Activity

**Figure 4:**
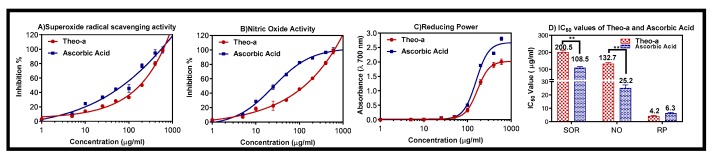
Dose-response inhibition of (**A**) superoxide radical activity, (**B**) nitric oxide activity, and of (**C**) reducing power of pheo-a and ascorbic acid. The half maximal inhibitory concentrations (IC_50_) for pheo-a and ascorbic acid (**D**). All the values are expressed as mean ± standard deviation; *n* = 3, and ** *p* < 0.01.

Usually, superoxides create hazardous hydroxyl radicals. Superoxide radicals (SORs) have been observed to initiate lipid peroxidation [31]. The antioxidant activity of pheo-a microparticles can be detected by its ability to inhibit the formation of blue nitro blue tetrazolium (NBT) [32]. Furthermore, the reduction in absorbance at 560 nm with antioxidant agents indicated the utilization of superoxide anions in the reaction mixture. The pattern of the dose-response curve of inhibition of SOR by pheo-a microparticles was similar to that of ascorbic acid with half maximal inhibitory concentration (IC_50_) 200.5 µg/mL and 108.5 µg/mL, respectively (Figure 4A). The conjugated cyclic structure, in addition to the carbonyl and amino groups, enabled pheo-a to scavenger superoxide radical and exhibited the antioxidant characters [33]. It was published that the pheo-a showed ability to inhabit the SOR activity with IC_50_ value 250 μg/mL [34].

### 2.5. Nitric Oxide Activity

All samples were incubated with sodium nitroprusside, which is able to produce nitric oxide free radicals. The nitric oxide free radical formation was more strongly decreased in concentration in a dependent manner by pheo-a, compared to the ascorbic acid, at 540 nm (Figure 4B). The inhibition of NO by pheo-a had a similar reaction as ascorbic acid with IC_50_ 132.7 µg/mL and 25.2 µg/mL, respectively. Cho et al., (2000) reported that pheo-a suppressed the nitric oxide production and inducible nitric oxide synthase (iNOS) mRNA expression and suggested that its antioxidant activity related to this mechanism of action [35].

### 2.6. Reducing Power Activity

Generally, the reducing ability is to measure the transformation of trivalent iron (Fe^3+^) to divalent (Fe^2+^). In this test, the yellow color was changed to different shades of green and blue colors as a result of reducing the power of pheo-a [36]. This color is also considered a significant indicator due to its potential antioxidant action. The results showed an increase in the absorbance to 680 nm, which indicates the reducing power of the pheo-a (Figure 4C). Furthermore, pheo-a has exhibited better reducing power activity than ascorbic acid with IC_50_ values of 4.2 µg/mL and 6.3 µg/mL, respectively. Liu et al. (2014) investigated the isolated compounds from leaves of *Nelumbo nucifera Gaertn. cv. Rosa-plena*, including pheo-a, by using three different methods: antiradical scavenging, metal chelating, and ferric reducing power assays. The results show that this compound has antioxidative activity [11].

### 2.7. Cytotoxic Assay

**Figure 5:**
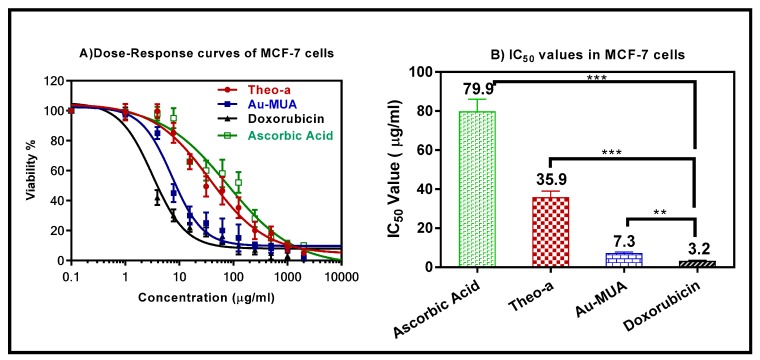
Dose-response curve (**A**) and half maximal inhibitory concentration (IC_50_) values (**B**) of pheophytin a (pheo-a) in Michigan Cancer Foundation 7 (MCF-7) cells in comparison to doxorubicin, ascorbic acid, and gold coated mercaptoundecanoic acid (AU-MUA). All the values are expressed as mean ± standard deviation; *n* = 3, *** *p* < 0.001, and ** *p* < 0.01.

After 24 h of exposure, the results show that pheo-a had no toxic effect when it was compared with the known cytotoxic agent, doxorubicin (Figure 5A). The IC_50_ values for pheo-a, ascorbic acid, gold nanoparticles, and doxorubicin were 35.9, 79.9, 7.3, and 3.2 µg/mL, respectively (Figure 5B). These results indicate that pheo-a has a less cytotoxic effect with moderately significant antioxidant activity, suggesting that the pheo-a can be used as an antioxidant agent with a broad dose, without having a harmful effect on viable cells.

## 3. Materials and Methods

### 3.1. Materials

Ethyl cellulose (EC), nitric oxide, and ascorbic acid were purchased from Sigma Chemical Co., (California, CA, USA). Methylcellulose (MC) was purchased from Dow Chemical Fluka (Greifensee, Swizerland). Chloroform (CHCl_3_), dichloromethane (DCM), calcium chloride (CaCl_2_) potassium chloride, tween 80, hydrochloric acid (HCl), sodium hydroxide, (NaOH), acetic acid (CH3COOH), and potassium dihydrogen ortho-phosphate (KH_2_PO_4_) were purchased from Elnasr Pharmaceutical Chemical Co. (Abu-Zaabal, Cairo, Egypt). All other chemicals and analytical reagents were of analytical grades and used as received.

### 3.2. Plant Materials

The plant material was collected in October 2016, from the flat arid high salted area near the main campus of Qassim University (GPS; 26°20′24.1″N 43°45′12.0″E). The plant was identified by Prof. Ahmed El-Oglah, department of biological sciences, Yarmouk University, Irbid, Jordan. A voucher specimen of the plant, under a number of 78, was deposited at the herbarium of the College of Pharmacy, Qassim University. The shaded dried herb was ground as a coarse powder for further work.

### 3.3. Extract Preparation

The powdered plant material (600 g) was extracted by Soxhlet continuous extractor [37] using petroleum ether, chloroform, ethanol, and water, in sequence with 10 refluxes each. The extracts were subjected to complete dryness using a rotary evaporator under reduced pressure and low temperature. The obtained extracts were stored at −20 °C for further work.

### 3.4. Isolation and Identification of Pheo-a

The chloroform extract was subjected to chromatographic isolation techniques in order to isolate its constituents. One gram of the total chloroform extract was subjected to a silica gel chromatography column using *n* hexane: ethyl acetate in a sequence ratio from 100:0 to 70:30. Five fractions were obtained: A, B, C, D, and E. All fractions were investigated for the presence of chlorophylls by thin layer chromatography (Silica gel 60, UV_254_, Merck) using *n* hexane: ethyl acetate (90:10 ratio) as mobile phase. Fraction A (250 mg) was subjected to Sephadex LH-20 column using methanol as an eluent to isolate two compounds in a pure form; compound 1, an intensive dark green sticky mass (120 mg), and compound 2, a white amorphous powder (30 mg). Pure compounds have been investigated spectroscopically using IR, Thermo Nicolet iS50 FTIR (Thermo Fisher Scientific Co., Waltham, MA, USA). NMR spectra were recorded using a Bruker Ultra Shield (400 MHz, Bruker Avance, Germany), and deuterated chloroform (CDCl_3_) was used as a solvent. The spectral data of these compounds have been used to match the same structures reported previously. Compound 1 was elucidated as pheophytin-a [24,38] while Compound 2 was elucidated as β-sitosterol [39].

### 3.5. Preparation of Ethyl Cellulose Microparticles Incorporated with Pheo-a

Microparticles were prepared according to the previously reported method [40]. In short, 150 mg of pheo-a was accurately weighed and dissolved in 70 mL of a 2% *w/v* solution of EC in (CHCl_3_). The resulting solution was then emulsified into 250 mL of 1% *w/v* aqueous solution of methylcellulose in distilled water. Stirring was continued at room temperature (25 °C) to allow CHCl_3_ evaporation. This process continued until the odor of the CHCl_3_ disappeared; then, the microparticles produced were separated by filtration using a Whatman membrane filters nylon with a pore size of 1 μm, diam. 47 mm. The separated microparticles were washed using distilled water and HCl (0.1M, pH 1.2). The washed microparticles were dried in a desiccator over CaCl_2_ for 48 h.

### 3.6. Size and Weight of Ethyl Cellulose Microparticles

The diameter of microparticles was determined using a Schimadzu particle size analyzer and the average weight of 10 of microparticles was determined. The mean of five determinations was considered as the weight of 10 microparticles. The samples were adjusted to 25 °C and then subjected to a laser beam of 633 nm at a scattering angle of 90° using the Shimadzu master sizer [21,41]. All samples were carried out in aqueous solution. The results were calculated from the average of the three measurements, while each measurement was run 20 times (with a 10 s duration).

### 3.7. Fourier-Transform Infrared Spectroscopy (FTIR)

FTIR can be used to measure the functional groups of the polymers and drugs [21,42]. The FTIR spectrometer (BRUKER Company, OPTIK GmbH, Germany) was used to identify the compatibility of all materials together. FTIR can record if an interaction between the EC and pheo-a, which prevented the release of pheo-a from EC [15]. The samples were put in an FTIR instrument under a spectrum from 4000 to 400 cm^−1^ using attenuated total reflection Fourier transform infrared spectroscopy (ATR-FTIR).

### 3.8. Antioxidant Activity

For determining the antioxidant activity, superoxide anion, which is a weak oxidant, was used as a marker. Superoxide anion produces hazardous hydroxyl radicals, as well as particular oxygen; both provide oxidative stress [43]. The activity of the superoxide anion free radical was examined by the method of Robak and Gryglewski [44]. In short, we used 5.0 mL of Tris–HCl buffer, conc. of (16 mM, pH 7.8), holding 0.5 mL of nitro blue tetrazolium (NBT) (0.3 mM), 0.5 mL NADH (0.936 mM) solution. Furthermore, 1.0 mL microparticles containing pheo-a and 0.5 mL Tris–HCl buffer (16 mM, pH 8.0) were supplemented to produce the superoxide anion radicals. Moreover, the reaction was induced by the addition of 0.5 mL phenazine methosulfate (PMS) liquid (0.12 mM) to the combination, which was then incubated at 25 °C for 5 min, and the absorbance was measured at 560 nm against a blank sample [36]. Finally, the superoxide anion free radical amount was estimated using the following equation:

$$\mathrm{Superoxide}\;\mathrm{anions}\;\mathrm{free}\;\mathrm{radical}\;\%=\frac{\left(A0-A1\right)}{A1}\times 100$$

where *A*0 is the absorbance of the control distilled water and *A*1 is the absorbance of the microparticles containing pheo-a.

### 3.9. Nitric Oxide Free Radical Activity (NO)

When arginine was metabolized to citrulline with the aid of nitric oxide synthase, NO was produced in biological tissues [45,46]. The formation of the nitric oxide free radical activity was carried out by adding 2 mL of sodium nitroprusside solution (10 mM) to 0.5 mL of a phosphate saline buffer (pH 7.4) with 0.5 mL of the sample at different strengths (0.2–0.8 mg/mL). Additionally, the blend was incubated at 25 °C, followed by a removal of 0.5 mL of the incubated solution, which was mixed with 0.5 mL of Griess reagent ((1.0 mL sulfanilic acid reagent (0.33% in 20% glacial acetic acid at 25 °C for 5 min with 1 mL of naphthyl ethylenediamine dichloride (0.1% *w/v*)). Then, the absorbance was measured at 546 nm [34]. Finally, the nitric oxide radical inhibition amount was estimated using the following equation:

$$\%\;\mathrm{inhibition}\;\mathrm{of}\;\mathrm{NO}\;\mathrm{radical}=\frac{\left(A0-A1\right)}{A1}\times 100$$

where *A*0 is the absorbance of the control distilled water, and *A*1 is the absorbance of the microparticles containing pheo-a after the reaction has taken place with the Griess reagent.

### 3.10. Reducing Power Method (RP)

The reducing power activity was measured as described by Oyaizu et al. [47], where 2.5 mL of the phosphate buffer pH 7, (0.2 M) and 2.5 mL of K_3_Fe(CN)_6_ (1% *w/v*) were added to 1.0 mL of the formulated microparticles and dissolved in distilled water. Additionally, the developed mixture was incubated at 50 °C for 20 min, followed by adding 2.5 mL of trichloroacetic acid (10% *w/v*); the mixture was then centrifuged at 3000 rpm for 10 min. A volume of 2.5 mL from the upper layer of the solution was collected and mixed with 2.5 mL of distilled water and 0.5 mL of FeCl_3_ (0.1%, *w/v*). Finally, the absorbance was measured at 700 nm against the blank.

### 3.11. The Cytotoxicity Assays

The human breast cancer cell lines (MCF-7) were grown in standard Roswell Park Memorial Institute series 1640 (RPMI 1640) media (10% fetal calf serum (FCS), antibiotic) at optimum temperature and humidity conditions (37 °C, 5% CO_2_). For the cytotoxicity assay, 5 × 10^4^ cells/mL of MCF-7 cells were seeded in 96-well microplates and allowed to adhere for 2 hours. Several different concentrations (µg/mL) were prepared by suspending the formulated pheo-a microparticles in a culture medium and applying them to cells. Untreated cells and positive and negative controls were considered in the experimental design. The cells were incubated for 24 h, and then the MTT assay was applied by replacing the media with MTT solution and incubating it for 4 h. Then, the formed formazan crystal was dissolved in dimethyl sulfoxide (DMSO) [48]. The color density was measured with spectrophotometry at 540 nm:

$$Viability\;\%=\frac{A_{U}-A_{T}}{A_{U}}\times 100$$

where the *A_u_* and *A_T_* are the absorbance of untreated and treated cells, respectively.

### 3.12. Statistical Analysis

The data of the studied compounds were expressed as mean ± standard deviation (SD). The international business machines- statistical package for the social sciences (IPM SPSS) statistics version 22 was used for statistical analysis. Statistical analyses between the different variables were calculated according to the two tail T test, which was used to compare the concentration of the studied compounds. *p* < 0.05 was thought to be statistically significant.

## 4. Conclusions

In conclusion, in this study, the naturally occurring pheo-a was successfully isolated from *S. vermiculata* and formulated in EC microparticles to improve the physicochemical properties of its parent secondary metabolite to enhance its bioavailability. The formulated microparticles have potent antioxidant properties and noteworthy cytotoxic activity of pheo-a compared to standard agents, providing good evidence that pheo-a can be used as an antioxidant agent in large doses without harmful effects to human cells.

## Supplementary Materials

The NMR and FTIR spectra for pheo-a and compound 2 are available online.
