# Supplementary material for: Formulation of Ethyl Cellulose Microparticles Incorporated Pheophytin A Isolated from Suaeda vermiculata for Antioxidant and Cytotoxic Activities
Source: Molecules. 2019 Apr 17;24(8):1501. doi: 10.3390/molecules24081501 (PMC6514815; doi:10.3390/molecules24081501)
Supplement: Supplementary file 1 [file molecules-24-01501-s001.pdf]

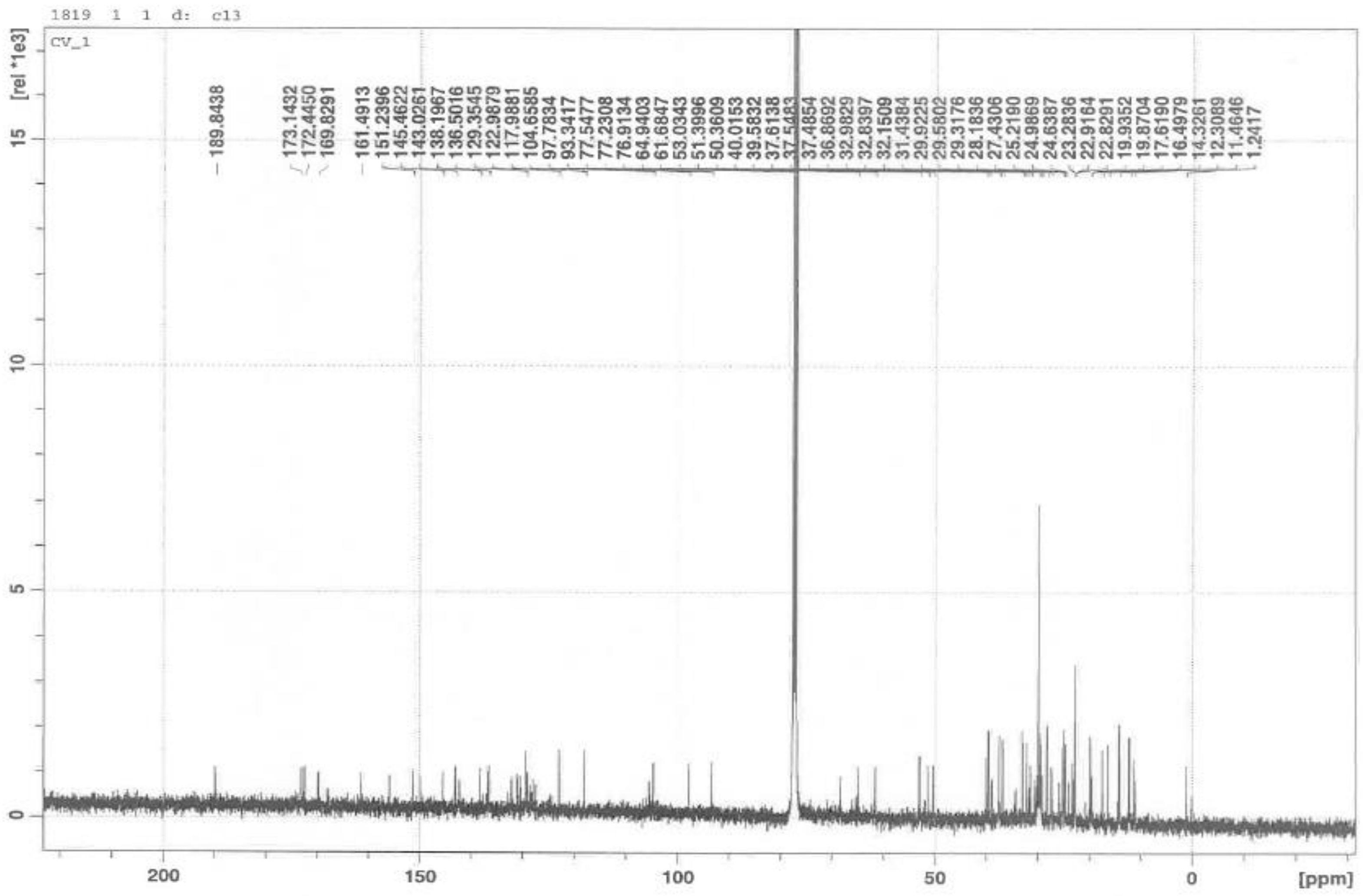

Figure 1: ^{13}C-NMR spectrum of Pheo-a

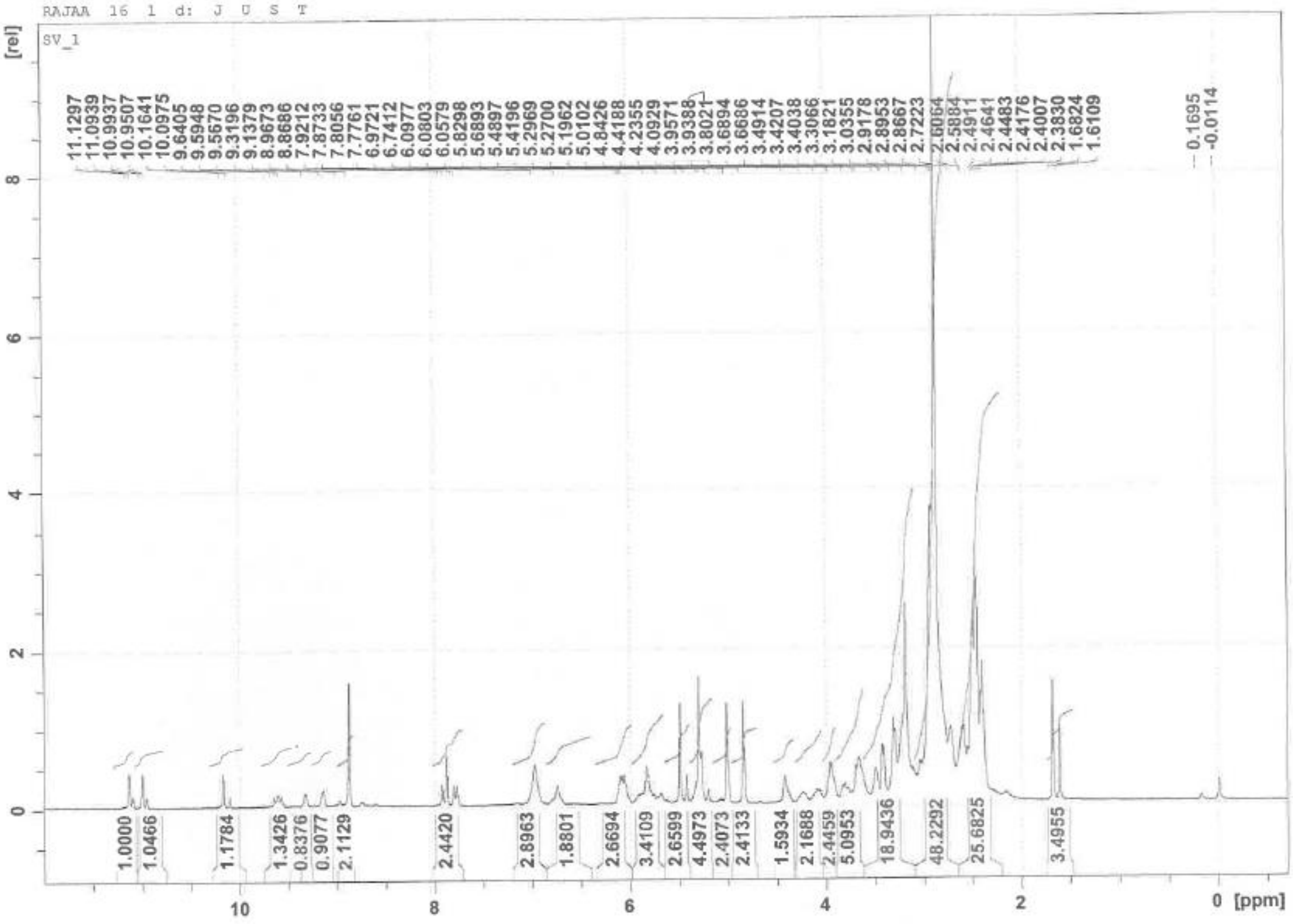

Figure 2:[1]**H-NMR specrum of Pheo-a**

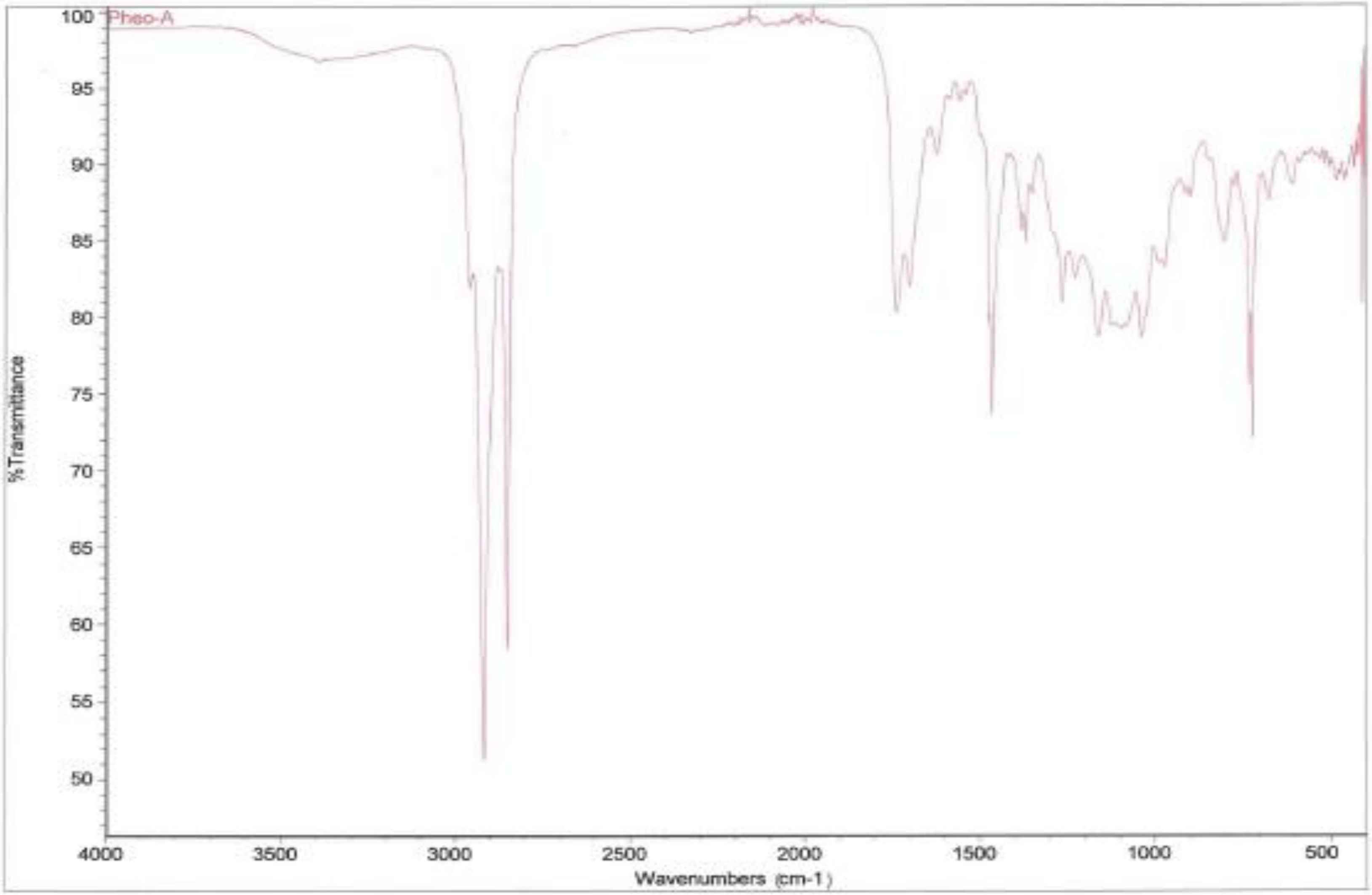

Figure 3: FTIR spectra of pheo-a

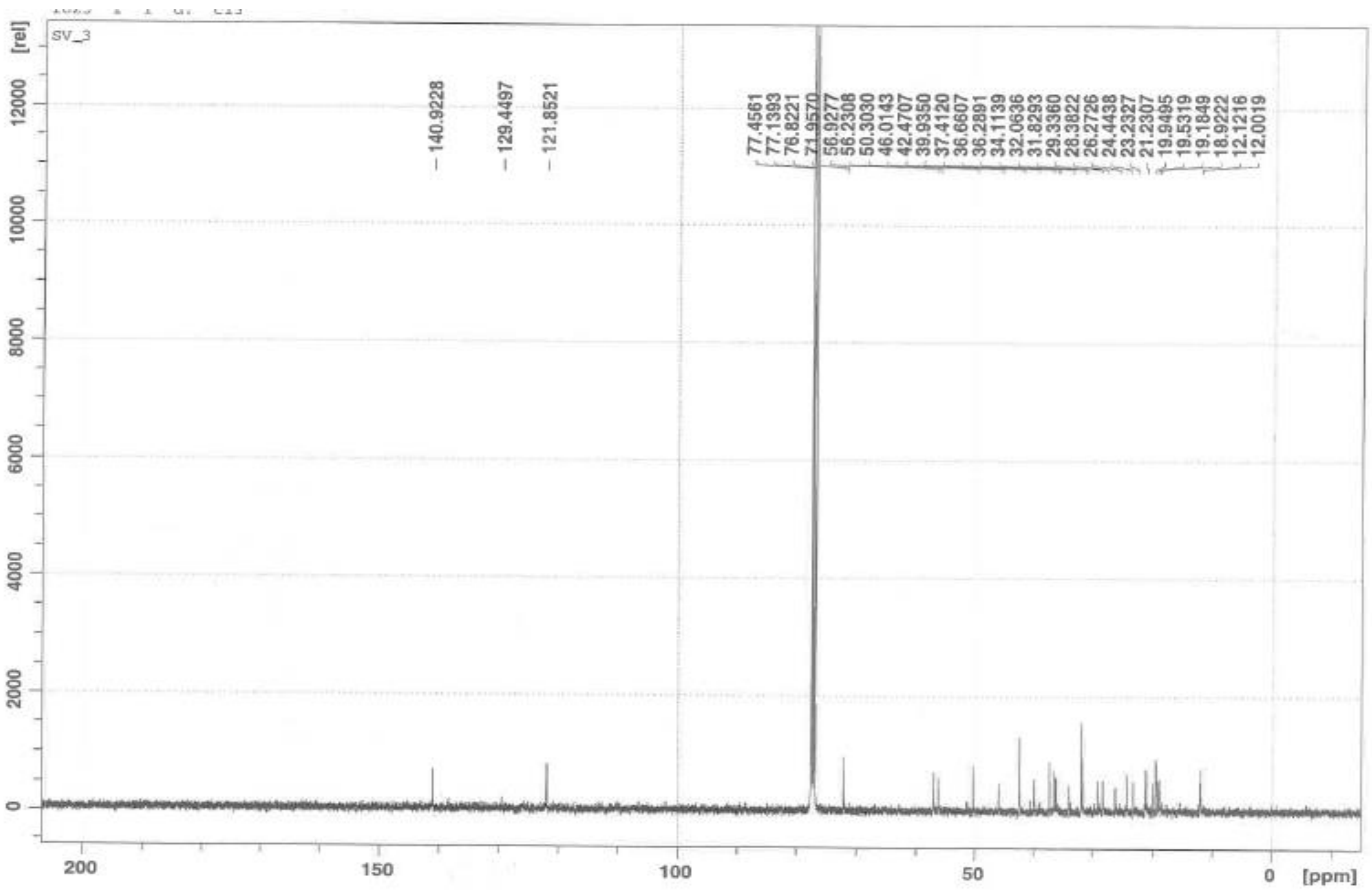

Figure 4: ^{13}C-NMR of compound 2

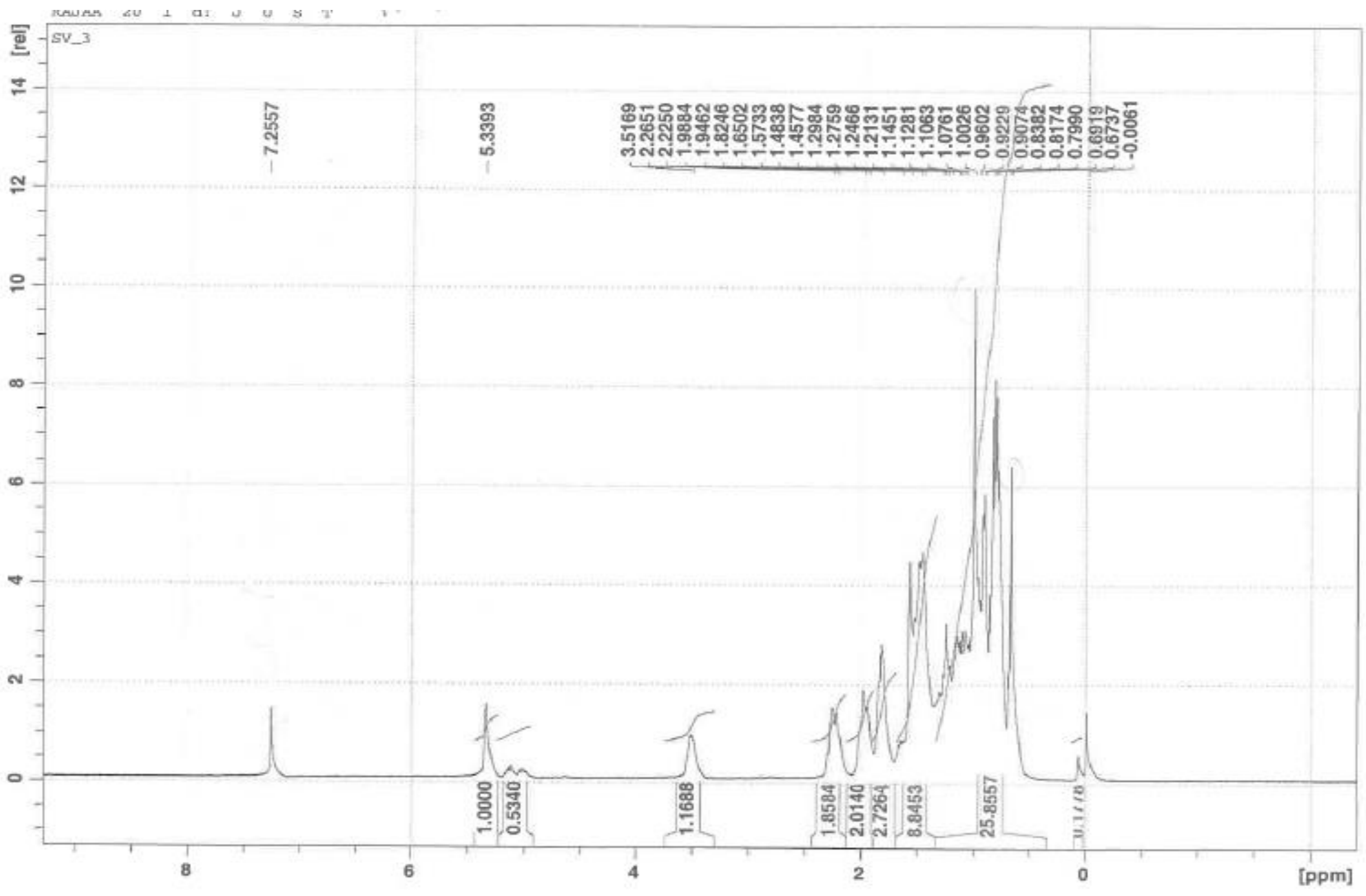

Figure 5: ^{1}H-NMR spectrum of compound 2

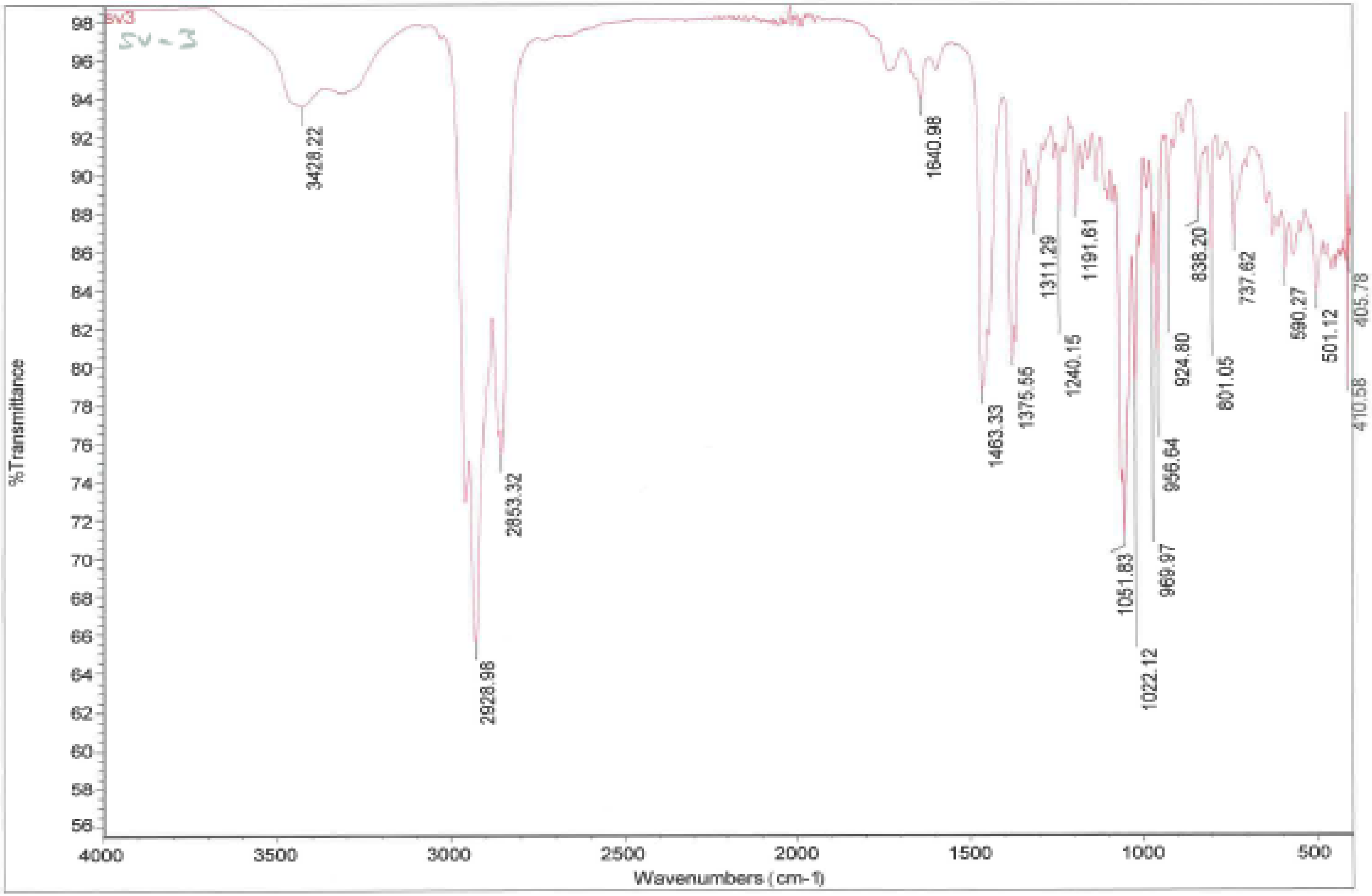

Figure 6: FTIR spectrum of compound 2
